# Auditory context-dependent distraction by unexpected visual stimuli

**DOI:** 10.3758/s13423-024-02527-y

**Published:** 2024-06-03

**Authors:** Fabrice B. R. Parmentier, Michael English, Murray T. Maybery

**Affiliations:** 1 Department of Psychology, University of the Balearic Islands, Ctra. De Valldemossa, Km 7.5, Palma de Mallorca, Balearic Islands Spain; 2 Balearic Islands Health Research Institute (IdISBa), Palma, Spain; 3 School of Psychological Science, University of Western Australia, Perth, Australia

**Keywords:** Attention, Distraction, Oddball, Environmental context

## Abstract

Research findings indicate that when a task-irrelevant stimulus feature deviates from an otherwise predictable pattern, participants performing a categorization task exhibit slower responses (deviance distraction). This deviance distraction effect reflects the violation of the sensory predictions generated by the cognitive system. In this study, we sought to examine for the first time whether these predictions can be incidentally modulated by the auditory environment. Participants categorized the duration (short vs long) of a colored shape (red square or blue circle) while instructed to disregard the stimulus’ visual features and the sound played in the background (two distinct chords played by different instruments). While the two visual stimuli shapes were equiprobable across the task, one was highly likely (p=.882) and the other rare (p=.118) in one auditory context and vice versa in the other context. Our results showed that participants were significantly slower in the duration judgement task whenever the stimulus was unexpected within a given auditory context (context-dependent distraction), and that the reset of their sensory predictions was completed upon the trial following a change of context. We conclude that object features and environmental context are processed in relation to each other and that sensory predictions are produced in relation to the environmental context, evidencing the first demonstration of auditory context-dependent modulation of attention.

## Introduction

In many situations, filtering out task-irrelevant stimuli contributes to efficient performance. However, blocking out such stimuli entirely would not be adaptive. Indeed, stimuli irrelevant for the task at hand may nevertheless convey information that is of potential importance outside that task. For instance, a reader engrossed in this article may be inclined to ignore distractors but would benefit from promptly detecting the unexpected occurrence of a fire alarm and adjusting their behavior accordingly. The involuntary detection of unexpected stimuli is therefore an adaptive counterweight to selective attention. Extensive research has documented the existence of neurocognitive mechanisms responding to stimuli contravening sensory predictions and leading to the involuntary capture of attention (Escera et al., 1998; Parmentier, 2014; Schröger, 1996, 2007; Schröger & Wolff, 1998; Wessel & Aron, 2013; Winkler, 2007). While adaptive, these mechanisms can, however, result in a temporary reduction in task performance (distraction). Here, we study distraction by the violation of sensory predictions. More specifically, we explore for the first time the extent to which these predictions are conditioned by the auditory environmental context.

Research shows that unexpected deviations in a sequence of otherwise predictable stimuli trigger the capture of attention, an involuntary orientation response, and lead to a decline in task performance (e.g., Parmentier, 2014), as revealed by specific electrophysiological responses (e.g., Schröger, 1996, 2005). Behavioral distraction follows a temporary inhibition of motor actions (Parmentier, 2016; Vasilev et al., 2019, 2021, 2023; Wessel, 2017; Wessel & Aron, 2013; Wessel & Huber, 2019) and the involuntary allocation of attention towards and away from the distractor (Parmentier et al., 2008; Parmentier & Gallego, 2020; Schröger, 1996; Weise et al., 2023). This effect arises from the violation of expectations rather than the base-rate probability of the distractors (Coy et al., 2022; Horváth & Bendixen, 2012; Parmentier et al., 2011; Parmentier & Hebrero, 2013; Schröger et al., 2007; Sussman et al., 2003), and is observed in categorization, go/no-go, visual matching, serial recall, gap detection, and duration discrimination tasks (Bendixen et al., 2010; Berti & Schröger, 2001; Escera et al., 1998; Hughes et al., 2005; Körner et al., 2017; Li et al., 2013; Pacheco-Unguetti & Parmentier, 2014, 2016; Röer et al., 2018; Vachon et al., 2017; Volosin et al., 2017; Volosin & Horváth, 2020). In summary, surprising stimuli capture attention, lead to behavioral distraction, and do so across different tasks and sensory modalities (Gerten & Topolinski, 2019; Parmentier et al., 2018; Wessel et al., 2012, 2016).

One fundamental aspect of deviance distraction relates to the conditions under which a task-irrelevant stimulus conveys surprise. While unexpected stimuli are typically defined with reference to a repetitive or predictable sequence of stimuli, the concept of context can be more broadly defined to include an aspect typically ignored in attentional research: The environmental context, defined as the “incidental information about the environment in which the focal information is processed” (Isarida & Isarida, 2010, p. 2399). To use an analogy: In a park in Paris, coming across a live penguin waddling about would be surprising, while spotting a squirrel would not. In contrast, in Antarctica, the sight of a penguin would be expected, but that of a squirrel scurrying along the ice would not.

Elements of the environmental context, although task-irrelevant, can be processed alongside task stimuli and modulate cognitive functioning. For example, in the field of memory, free recall performance is partly contingent upon whether encoding and retrieval take place in the same or different locations (Bjork & Richardson-Klavehn, 1989; Godden & Baddeley, 1975; Smith & Vela, 2001). These effects have also been documented using recognition tasks (Dalton, 1993; Macken, 2002) and in the realm of eyewitness testimony (Krafka & Penrod, 1985; Malpass & Devine, 1981; Smith & Vela, 1992). Research also indicates that memory interference from irrelevant stimuli is attenuated if learned in an environment different from that of the to-be-remembered items (Bilodeau & Schlosberg, 1951; Dallett & Wilcox, 1968; Greenspoon & Ranyard, 1957; Smith & Vela, 2001). While much less researched, attentional functioning processes can also be modulated by the environmental context. For instance, task-irrelevant background images (black and white forest versus city scene) can bias participants’ visual search strategy (Cosman & Vecera, 2013).

We recently demonstrated that the distraction yielded by task-irrelevant sounds is mediated by their integration with the environmental context (Parmentier et al., 2022). In two experiments, participants categorized left and right arrows while disregarding extraneous auditory stimuli and background images depicting either forest or urban scenes. Sounds A and B were presented with probabilities of 0.882 and 0.118 in the forest environment, respectively, and with the inverse probabilities in the urban setting. Since the two contexts were equiprobable across the task, neither sound represented a deviant auditory stimulus at the task level, but each did so within a specific context. The results were clear: Participants exhibited a significant lengthening of response time in the visual task following the presentation of the sound that was unexpected in the current context, indicative of context-dependent distraction. This pattern of results was observed when the forest and city contexts were defined by a single image each across the task, as well as when they consisted of varying pictures of forests and cities. In a follow-up study (Parmentier, Gallego & García-López, submitted), similar results were reported using a unimodal task in which participants categorized the duration (short vs. long) of a visual stimulus (blue circle or red square) presented against background images (forest or city scenes). From these studies, we concluded that irrelevant stimuli are processed in conjunction with the environmental context, whether they are presented within the same sensory modality or across modalities, and that sensory predictions are context-dependent.

Distraction by unexpected sounds is typically interpreted as the result of the mismatch between the incoming stimulus and a sensory prediction computed from such events (Korka et al., 1992; Schröger & Roeber, 2021; Winkler, 2007), in line with the general view that our cognitive system generates predictive models (Friston, 2005, 2010). Our recent findings (Parmentier et al., 2022, Parmentier et al., submitted) certainly suggest that predictions are computed based on an integration of the environmental context and the task stimuli (task-relevant or not).

In attention studies, context has been exclusively implemented as background images (Cosman & Vecera, 2013; Parmentier et al., 2022). In contrast, context-dependent memory effects have been reported with different types of context—for example, immersive situations, whether natural (Godden & Baddeley, 1975) or virtual (Shin et al., 2021); background color (Dulsky, 1935; Macken, 2002); chewing gum (Baker et al., 2004; Miles & Johnson, 2007; Overman et al., 2009); posture (Rand & Wapner, 1967), or background music (Balch et al., 1992; Fagen et al., 1997; Pescara-Kovach et al., 2000; Smith, 1985). Comparatively, the auditory environment has scarcely been studied, even though sound is a ubiquitous feature of everyday life situations. A handful of studies found a significant memory advantage when items were encoded and retrieved with the same background music compared with a different or no auditory background (Balch et al., 1992; Fagen et al., 1997; Pescara-Kovach et al., 2000; Smith, 1985). With respect to attention, research on the effect of background sound has centered on its modulation of arousal or valence (Burkhard et al., 2018; Cloutier et al., 2020) or its potential entrainment value (e.g., Bolger et al., 2013). There exists, to our knowledge, no study examining the impact of sound as an environmental context on attentional distraction.

In this study, we examined for the first time the potential integration of task-irrelevant stimulus features and environmental context in the form of task-irrelevant background sound. Participants judged the duration of a visual stimulus while instructed to ignore its visual aspect (shape and color) and the auditory context. Two contexts were used, each corresponding to a musical chord played by a distinct instrument. The two visual stimuli were used equiprobably across the task but not within each of the two contexts: one was highly probable in one context but unexpected in the other, and vice versa. Several hypotheses can be entertained. Under a strict context-independent hypothesis, performance should be identical for both stimuli across the two contexts because none of them constitutes a deviant stimulus at task-level. In contrast, under the context-dependent hypothesis, task-irrelevant features of the target stimuli should be appraised in relation to the auditory context in which they occur, such that a stimulus should constitute a deviation whenever it violates sensory predictions within that context and therefore lengthen response times compared with a predictable stimulus.

## Method

### Participants

Forty-one participants ages 18–53 years (*M* = 21.07 years, *SD* = 6.48) took part in this study (25 were female). Two participants were lefthanded. All were undergraduate psychology students at the University of Western Australia who took part in the study in exchange for course credit.

### Material and stimuli

Two musical chords were recorded using a Roland HP-605 digital piano. The first chord was B minor seventh flat five (Bm7b5, containing the following notes: B1, B2, A3, B3, E4, F4) recorded using the WarmPad sound sample (a rounded and warm synthesizer sound, defined as a soft, elongated sound typically designed to “pad out” a track or section of instrumentation and used to create a sense of atmosphere). The second chord was F major (containing the notes F2, F3, C4, F4, A4) recorded with the Symphonic String sample (a mix of violin, viola, cello and bass). These sounds were selected to be distinct both harmonically, in terms of timbre, and for producing sustained sound. The chords were played in the same manner, with all keys pressed simultaneously and with the same force. Peak amplitude was normalized to −0.1 dB using the Audacity software (thereby keeping the sound’s maximum amplitude peak 0.1 dB below the level where there would be distortion or clicks).

The task was programmed using Psychology Software’s E-Prime 3.0 software and was executed on a PC computer equipped with a 24-in. screen. Auditory stimuli were delivered diotically through headphones, at an intensity of approximately 70 dB SPL.

### Procedure

The participants’ task was to categorize the duration of a visual stimulus (a red square or a blue circle) as short (200 ms) or long (800 ms). These two durations were selected based on prior work (Leiva et al., 2015). Participants were asked to focus on the stimulus’ duration irrespective of its visual aspect, and to ignore the sounds presented through the headphones.

**Fig. 1:**
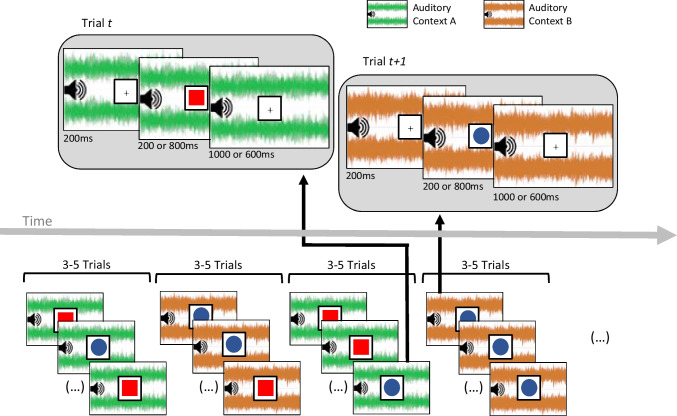
Schematic illustration of the task. The bottom part of the figure illustrates successive runs of 3 to 5 trials sharing the same auditory context. The upper part illustrates the timeline of the specific individual trials signaled by black arrows (see the Method section for a detailed description of the design and procedure). Participants were required to discriminate between the shorter and longer durations by pressing corresponding response keys (counterbalanced across participants) and ignoring the visual features of the target stimulus. They were also asked to ignore the auditory context (A or B). Runs of 3 to 5 trials of the same auditory context were presented in sequence (note that to avoid visual overcrowding of this figure, we display 3 pictures per run). (Color figure online)

Each trial began with the onset of one of the two background sounds, hereafter referred to as Auditory Context A or B (the F major chord played by symphonic strings or the Bm7b5 chord played by synthesized WarmPad, respectively, counterbalanced across participants), and a black fixation cross displayed within a 100 × 100-px white square (framed in black) at the center of the screen. These stimuli remained present throughout the trial, except when the target stimulus temporarily replaced the fixation cross (as described below). Following an interval of 200 ms, one of two visual stimuli (a red square or a blue circle) was presented within the central frame, for 200 or 800 ms. Participants were instructed to respond by pressing the V or M key using their left or right index fingers, respectively (the mapping of the keys to the short/long responses was counterbalanced across participants). Following the offset of this stimulus, the fixation cross returned during a further interval of 1,000 or 600 ms, respectively (such that the total interval between the visual stimulus’ onset and the end of the trial was fixed to 1,200 ms in all trials). Upon completion of this interval, the next trial began automatically. A schematic is presented in Fig. 1.

The choice and arrangement of the auditory context and target stimuli followed specific rules. Across the task, the two auditory contexts were used equiprobably in successive runs of trials of 3 to 5 consecutive trials, creating a total of 1,216 test trials. That is, there were 3–5 trials with one auditory context, then 3–5 trials with the other auditory context without any change in the continuity of trials, and so on in alternation. Across these two auditory contexts, both visual stimuli (red square and blue circle) were presented equally often. However, they were not equiprobably presented within each of these two contexts. One shape (e.g., red square) was more likely in one context (e.g., strings) than the other (e.g., blue circle): *p* = .882 and *p* = .118, respectively. These probabilities were reversed in the other auditory context (e.g., WarmPad). Hence, while each visual stimulus was presented in half the trials across the task, it constituted a standard stimulus in one context and a deviant stimulus in the other. The allocation of the visual stimuli (red square and blue circle) to these conditional probabilities was counterbalanced across participants. Within a run of trials of the same context, visual stimuli constituting a deviant within that context were never presented on the first trial of a run. Finally, only half of the runs of trials of a specific context involved a trial with a deviant stimulus (to avoid expectation effects). Each participant received a distinct pseudo-randomized set of trials based on the rules described above. The 1,216 test trials were divided into 4 blocks of 304 trials lasting about 7 minutes each (participants were allowed to pause for a short rest between blocks).

Before participants completed the 4 blocks of test trials, they performed a block of 20 practice trials in which no background sound was presented. Feedback was provided after each response in the form of text appearing for 1,500 ms after the participant’s response. This feedback indicated whether the response was correct, incorrect, or urged participants to respond faster if they failed to respond. This feedback was not presented in the rest of the experiment.

### Analyses

We analyzed the mean proportion of correct responses and the mean response times (RTs) for correct responses. RTs were measured from the moment of presentation of the critical information—that is, 200 ms into the visual stimulus’ presentation (i.e., the shortest duration, such that stimuli remaining beyond that point constituted long stimuli). Effect sizes are reported as partial eta-square values for *F* tests, and as Cohen’s *d*_az_ for within-participant comparisons and d for between participants comparisons (Lakens, 2013). All *t* tests were two-tailed, except where explicitly stated otherwise. In addition to frequentist statistics, we also report the Bayes factor (BF_10_) to assess the credibility of the experimental hypothesis relative to that of the null hypothesis given the data. Values below 1/3 are considered as substantial to strong support for the null effect, while values above 3 are regarded as substantial to strong support for the presence of an effect (Jarosz & Wiley, 2014; Jeffreys, 1961). Trials with response times shorter than 100 ms were treated as anticipations and excluded from the analysis. An a priori power analysis was carried out based on the smallest effect size of the Context × Stimulus interaction we reported in a similar study, only where deviance was carried by an auditory distractor (Parmentier et al., submitted). For an effect size of *d* = 0.829, a Type I error probability of .05 and a power of .95, the minimum sample size is 21. Our sample size was larger.

## Results

### Deviance distraction as a function of auditory context

The mean proportions of correct responses (Table 1) were analyzed using a 2 (auditory context: strings vs. WarmPad) × 2 (stimulus: A vs. B) analysis of variance (ANOVA) for repeated measures. There was no main effect of context, *F*(1, 40) = 1.467, *MSE* = .000641, *p* = .233, ${\eta }_{p}^{2}$ = .035, *BF*_*10*_ = 0.281; stimulus, *F*(1, 40) = 0.021, *MSE* = .000869, *p* = .959, ${\eta }_{p}^{2}$ < .001, *BF*_*10*_ = 0.169; or interaction between these factors, *F*(1, 40) = 0.277, *MSE* = .00134, *p* = .602, ${\eta }_{p}^{2}$ = .007, *BF*_*10*_ = 0.255.

**Table 1:** Mean proportions of correct responses as a function of the auditory context and the visual target stimulus (A, B)

		Stimulus	
		A	B
Auditory context	Strings	.894 (0.011)	.891 (0.010)
	WarmPad	.896 (.009)	.898 (0.011)

*Note*. Numerical values within brackets represent one standard error of the mean

**Fig. 2:**
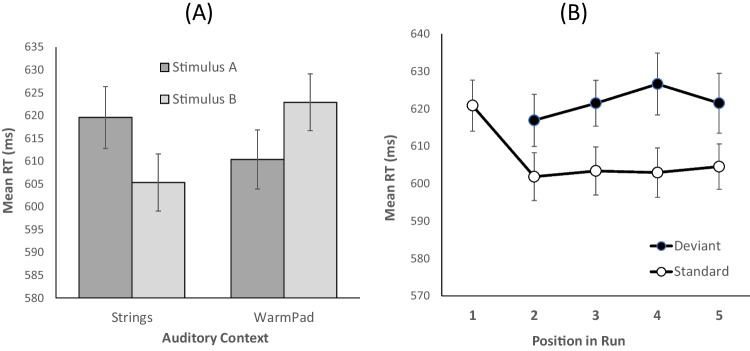
**(A)** Mean response times as a function of the auditory environment (Strings, WarmPad) and the visual stimulus (A, B). In the Strings context, stimuli A and B were presented with probabilities of.118 and .882, respectively. These probabilities were reversed in the WarmPad context. **(B)** Mean response times for context-specific standard and deviant stimuli as a function of the position of the trial within a run of trials sharing the same context. Error bars represent one standard error of the mean

For RTs, the main effect of context was significant, *F*(1 ,40) = 4.425, *MSE* = 163.2, *p* = .042, ${\eta }_{p}^{2}$ = .100, but the *BF*_*10*_ was inconclusive (0.624). The main effect of stimulus was not significant, *F*(1, 40) = 0.255, *MSE* = 119, *p* = .617, ${\eta }_{p}^{2}$ = .006, *BF*_*10*_ = 0.175. Critically, the interaction between these factors was significant, *F*(1, 40) = 21.853, *MSE* = 316.2, *p* < .001, ${\eta }_{p}^{2}$ = .353, *BF*_*10*_ = 440386.608 (see Fig. 2A). Within each context, RTs were significantly longer for the least frequent stimulus relative to the most frequent stimulus: Stimulus A relative to Stimulus B in the strings context, *t*(40) = 4.73, *p* < .001, *d*_*az*_ = 0.739 (95% CI [0.389, 1.081]), BF_*10*_ = 781.6), and Stimulus B relative to Stimulus A in the WarmPad context, *t*(40) = 3.46, *p* < .001, *d*_*az*_ = 0.540 (95%CI [0.209, 0.865]), BF_*10*_ = 23.8.

### Temporal dynamics across same context runs

We analyzed RTs across runs of trials involving the same auditory context as a function of whether the visual stimulus constituted a standard or a deviant stimulus in that run. Such runs contained 3 to 5 trials and always started with a trial in which the stimulus presented corresponded to the standard stimulus within the current context. Our aim was to determine how quickly the cognitive system reconfigured its sensory predictions upon a change in auditory context. We conducted this analysis in two steps. First, we compared the mean performance in the first trial of a run (always standard; STD1) with performance in the second trial (standard or deviant; STD2 or DEV2, respectively). Second, RTs in Trials 2 to 5 of a run were analyzed using a 2 (stimulus condition: standard vs. deviant) × 4 (position: Trials 2–5 within a run) ANOVA for repeated measures.

The mean RT on the first trial (STD1) was significantly longer than that on the second standard trial, STD2; *t*(40) = 8.399, *p* < .001, *d*_*az*_ = 1.312 (95% CI [0.888, 1.726]), *BF*_*10*_ = 46003064.602, but similar to that on the deviant trial in second position, DEV2; *t*(40) = 1.124, *p* = .268, *d*_*az*_ = 0.176 (95% CI [−0.134, 0.483]), *BF*_*10*_ = 0.303. Across Positions 2 to 5, responses were slower for deviant than for standard trials, *F*(1, 40) = 31.758, *MSE* = 877.431, *p* < .001, ${\eta }_{p}^{2}$ = .443, *BF*_*10*_ = 1855241841.579. There was no main effect of position, *F*(3, 120) = 0.851, *MSE* = 487.243, *p* = .469, ${\eta }_{p}^{2}$ = .021, *BF*_*10*_ = 0.032, or the interaction between condition and position, *F*(3, 120) = 0.612, *MSE* = 460.346, *p* = .608, ${\eta }_{p}^{2}$ = .015, *BF*_*10*_ = 0.060. In sum, a stimulus acting as a deviant in a run of trials of the same context still exerted distraction on the very first trial following a change of context (i.e., when it now constituted a standard stimulus; see Fig. 2b) but not beyond.

To examine these results further and disentangle the effect of context-dependent deviance from the visual change of stimulus, we conducted a secondary analysis where we contrasted position in the run (1 vs. 2–5) and whether the stimulus differed visually from, or was identical to, that presented on the previous trial. The rationale for this analysis is as follows: If the increase in reaction time (RT) observed in the first trial following a change of context reflected a perceptual change (even in part), we would expect to see a larger difference between Position 1 and Positions 2–5 in trials involving a change of stimulus compared with trials with no such change. In other words, the interaction between position and stimulus change should be significant. Conversely, if stimulus change does not contribute to the effect we previously reported, no such interaction should be observed. The main effect of position was significant, *F*(1, 40) = 10.628, *MSE* = 318.763, *p* = .002, ${\eta }_{p}^{2}$ = .210, BF10 = 17.045, as was that of stimulus change, *F*(1, 40) = 33.508, *MSE* = 227.379, *p* < .001, ${\eta }_{p}^{2}$ = .456, BF10 = 11338.328. Importantly, these two factors did not interact, *F*(1, 40) = 1.563, *MSE* = 246.210, *p* = .218, ${\eta }_{p}^{2}$ = .038, BF10 = 0.183. Hence, the data show that the increase in RT on the first position of a run is not explained by the change of visual stimulus and instead is due to the change in context.

## Discussion

We examined whether task-irrelevant stimuli can acquire the condition of deviant stimuli and exert behavioral distraction dependent upon their integration with the auditory context. The results showed that performance in a visual duration judgement task is negatively affected by changes in the stimulus’ visual features to the extent that these changes were unexpected within the current auditory context. The task-irrelevant features of the target stimuli (shape and color) were processed in integration with the auditory context. As a result, the two visual stimuli, though equiprobable across the task, acquired the status of deviant or standard stimuli depending on the current environmental context. An analysis of the temporal dynamics of this effect demonstrated that a change of auditory context is not instantaneously accompanied by a resetting of the participants’ sensory predictions. That is, a stimulus that constituted a deviant stimulus immediately prior to a change in auditory context maintained its full distractive impact on the first trial following the change (i.e., when it now constituted a standard stimulus). This inertial effect was only observed on that first trial following a change of context, however. These findings reveal that participants processed the relevant stimulus information (duration) in relation to its task-irrelevant features (shape and color) and the auditory background. To our knowledge, this constitutes the first demonstration of an auditory context-dependent modulation of attentional distraction.

Our results are in line with those found in experiments where the context consisted of background images (Parmentier et al., 2022, submitted). More importantly, they extend the relatively scarce work on the role of auditory environmental contexts. Some reported enhanced memory performance when the same piece of background music was played at encoding and at test, whether in free recall (Smith, 1985), immediate unexpected recognition (Pescara-Kovach et al., 2000), delayed surprise recall (Balch et al., 1992; Fagen et al., 1997), or incidental learning tests (Fagen et al., 1997; Pescara-Kovach et al., 2000). The present work constitutes the first study showing that the auditory context modulates distraction by unexpected changes in the visual modality. Because our participants categorized the duration of visual stimuli with no intention of encoding information for later recall, our findings suggest that the learning of associations between task-relevant and task-irrelevant information, as well as the predictions generated based on such associations, occurs involuntarily. It is therefore in line with the modulation of attentional mechanisms by visual contexts (Anderson, 2015; Cosman & Vecera, 2013; Parmentier et al., 2022).

Our results also suggest that an auditory context can be defined with stimuli as simple as single chords played by distinct instruments. Prior work on memory used music (e.g., jazz or classical pieces) conveying more complex information, including melodic and harmonic cues, which may elicit deeper processing. Furthermore, musical stimuli have the potential of inducing emotions and affect arousal, which in turn may potentially constitute contextual information encoded alongside the attended stimuli. While this may be especially effective in indirectly reinforcing links between successive items in memory, background music may also potentially provide a richer context and impact performance on categorization tasks like ours. On the other hand, one could argue that the simplicity and lack of dynamic variation of our stimuli may provide a more robust and stable context. We note that in prior work using oddball tasks involving visual contexts, the modulation of distraction by unexpected stimuli was stronger when each context consisted of a single picture as opposed to multiple pictures (Parmentier et al., 2022, submitted). The deviance distraction effect we observed in the present study is certainly not marginal, for its effect size across Positions 2 to 5 of a run of trials sharing the same context was large (*d*_az_ = 0.937) and in line with what we observed with a fixed visual context (Parmentier et al., 2022, Experiment 1, *d*_az_ = 1.116). Nevertheless, whether the strength of contextual modulation of distraction may vary with a more complex auditory context such as a musical piece remains an open question that could be addressed in future research.

One interesting aspect of our findings is that a change of auditory context did not instantaneously translate in the update of predictions by the participants’ cognitive system. This is consistent with what we observed using an identical task but where the environmental context consisted of fixed or varying pictures of forest or city scenes (Parmentier et al., submitted) as opposed to background sounds. Further research will be required to ascertain the exact nature of the mechanisms underpinning the resetting of predictions. We note however that this finding is reminiscent of the residual cost observed in task switching paradigms where the switch cost is only partially diminished by task cues or a long preparation interval, and task switching is not fully achieved until after a trial has been completed (Longman et al., 2014; Meiran, 1996; Meiran et al., 2000; Schneider, 2017). Explanations of this residual cost include (1) the notion of task-set inertia whereby positive and negative priming of the no-longer relevant and relevant task sets, respectively, exert interference in the early stages of a switch (e.g., Allport et al., 1994), and (2) the contention of an exogenous component of task switching that requires the presence of the stimulus for the switch to be completed (e.g., Rogers & Monsell, 1995; Rubinstein et al., 2001). Analogous mechanisms may be at play in our study. The short-lived inertia of distraction observed upon a change of context may reflect the time required to process the background information and carry out the resetting of predictions contingent upon this context. It may also be that predictions are only totally reset once the first stimulus has been processed following the change of context, thereby completing an integrative episode involving context, target and task-irrelevant information and the production of a response based on updated predictions.

In conclusion, our study reveals that individuals judging the duration of a visual stimulus also process irrelevant visual features of this stimuli, the auditory environmental context, and the association between the latter two. As a result, the auditory context generates sensory predictions that, if violated, result in behavioral distraction in the form of longer response times.

## Data Availability

The dataset generated and analyzed during this study is available on the Open Science Framework (https://osf.io/gk49u/). The study was not preregistered.

## Code availability

The task used is not openly published but can be obtained from the corresponding author upon request.
